# Ecotoxicological and Microbiological Risk Assessment of Groundwater from Dimba Cave, Democratic Republic of the Congo

**DOI:** 10.3390/ijerph21080962

**Published:** 2024-07-23

**Authors:** Daniel M. Mudinga, Archal M. Ngandote, John M. Kayembe, Séraphin N. Lusamba, Emmanuel K. Atibu, Fernando P. Carvalho, John Poté

**Affiliations:** 1 Faculté des Sciences, Université Pédagogique Nationale, Kinshasa XI B.P. 190, Democratic Republic of the Congojohn.pote@unige.ch (J.P.); 2 Department of Chemistry, Faculty of Science, University of Kinshasa, Kinshasa XI B.P. 190, Democratic Republic of the Congo; 3 Laboratório de Protecção e Segurança Radiológica, Instituto Superior Técnico/Campus Tecnológico Nuclear, Universidade de Lisboa, 2695-066 Lisboa, Portugal; 4 Department F.-A, Faculty of Science, Forel for Environmental and Aquatic Sciences, and Institute for Environmental Sciences, University of Geneva, 1211 Geneva, Switzerland

**Keywords:** Dimba Cave, heavy metals, microbial contamination, ecotoxicological risk

## Abstract

Dimba Cave is a large array of natural galleries in limestone mountains of the Democratic Republic of the Congo that contains highly valued pre-historic archaeological artifacts. The cave attracts a high number of tourists every year and is used by local populations as a water supply source. The main objective of the research undertaken in Dimba Cave consisted of assessing the quality of water and sediments from Dimba Cave ponds through evaluating contamination by heavy metals (15 elements analyzed, including As, Cd, Pb, and Hg) and by microbial populations (including *Escherichia coli* and total coliforms) in order to estimate the ecotoxicological risk to humans and to non-human biota. All water samples collected in the cave ponds showed very high metal concentrations exceeding the internationally recommended limits for drinking water, particularly for Cr, Mn, As, Pb, and Hg. Most sediment samples from cave ponds also displayed high heavy metal concentrations. The calculated pollution parameters, such as the enrichment factor (EF), and ecological risk parameters, such as the ecological risk index (Eri), indicated that the sediment may be toxic to aquatic biota. Furthermore, the microbiological analysis of pond waters indicated a widespread contamination with bacteria such as *Escherichia coli*, *Enterococcus* spp., total coliforms, and *Pseudomonas* spp., probably from anthropogenic and/or animal sources. Therefore, the consumption of Dimba Cave water as a drinking water represents a threat to public health. Urgent management measures should be enforced to protect public health and the cave ecosystem.

## 1. Introduction

Dimba Cave was discovered in the beginning of the 20th century, around 1920, and it was found to contain vast archeological artifacts of considerable interest to the investigation of pre-historic times of the region [1,2]. This cave is located in the territory of Mbanza-Ngungu, in the central part of the Democratic Republic of the Congo (DRC). Currently, the cave has high touristic frequency and because of the archaeological vestiges found therein, it has potential for being inscribed in the UNESCO World Heritage List. Therefore, the investigation and preservation of this unique Dimba Cave should be a priority.

Caves are common in limestone formations and are karstic structures carved by water. In general, the cave environment is dark, humid, and with minor temperature fluctuations, and, although offering a scarce supply of nutrients to the cave biota, provides habitats with stable conditions for many unique and endemic species [3,4,5,6,7]. The preservation and research on the fauna and flora of this uncommon environment is nowadays a priority in many countries (e.g., USA, Turkey, Slovenia).

Over time, many natural caves have been widely used by humans for different purposes, such as temporary shelter, residence, religious sanctuaries, extraction of natural resources, and tourism as show caves. Caves have also been used for disposal of household waste, especially in karst areas close to dwellings and, in the absence of regulations and suitable management, this has led to cave pollution and menace to this special environment [8,9].

Several studies on cave pollution have focused on contamination by heavy metals and invasive bacteria and, in particular, on fecal bacteria that have been used as an indicator of water and sediment contamination caused by waste from anthropogenic and animal origins [5,10,11,12,13]. These studies have shown the need for evaluating the concentration and distribution of heavy metals and microorganisms in cave waters and sediments in order to enable the assessment of ecotoxicological risks in touristic caves and to help their conservation [14,15,16,17].

The current investigation on Dimba Cave focused on the determination of 15 metallic elements in the water and sediments from cave ponds and on the microbiological analysis of cave water. This study is the first physicochemical and microbiological evaluation of water and sediments from Dimba Cave and is aimed at providing an assessment of the status of the cave environment and at identifying ecological threats as well as establishing the need for cave-management procedures.

## 2. Material and Methods

### 2.1. Description of the Study Area

**Figure 1:**
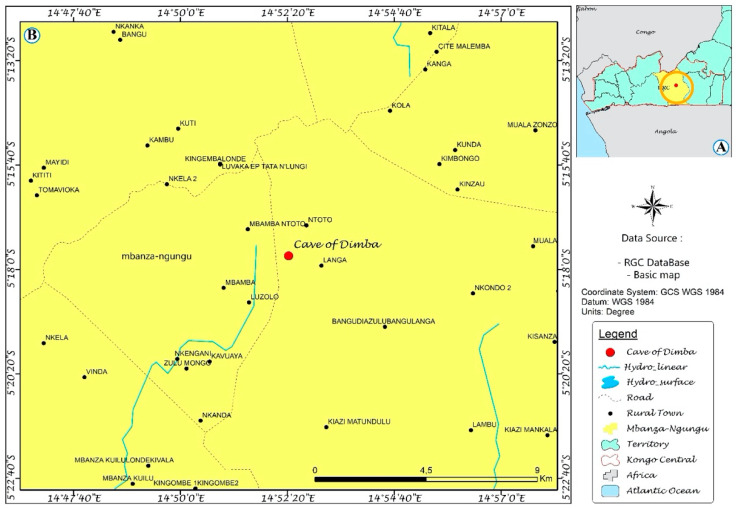
(**A**) Mbanza-Ngungu territory located in Kongo central province of the DR Congo, (**B**) location of the Dimba Cave (red dot).

Dimba Cave is an underground array of tunnels of natural origin carved by groundwater in limestone. The cave entrance is located 5 km south of the village of Mbamba Ntoto, in the Mbanza-Ngungu territory, DRC (geographic coordinates 05°17′42″ S and 014°52′23″ E). This cave spreads across a large part of the Cataracts district of DRC (Figure 1). A few meters into the cave entrance, there is a gallery of 6 m × 4 m on the left, arranged during colonization for meetings between settlers and villagers. Nearby, there is the tomb of an old chief of the Mbamba Ntoto village that is a place of ritual ceremonies by villagers.

The local population developed special beliefs about Dimba Cave and practice spiritual rites therein. Furthermore, the population consumes cave water based on the belief that it cures several diseases, hunts bats for food, and carries out artisanal mining of lime and bat guano from the cave. Meanwhile, the cave has also become a major touristic attraction of the region.

Since 1997, this cave has been on the indicative list of properties that the Democratic Republic of the Congo government wants to propose for inscription into the UNESCO World Heritage List [18]. So far, the cave is not protected by specific regulations, and access is unrestricted.

**Figure 2:**
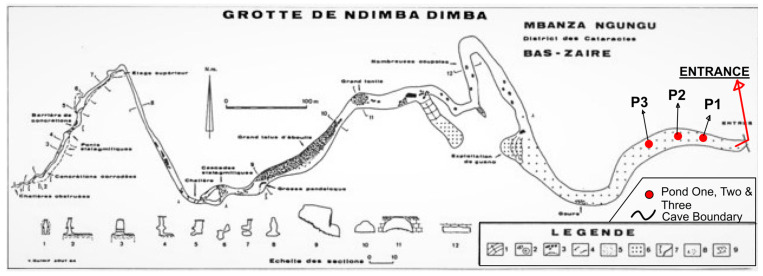
Dimba Cave plan (based on Quinif et al. [3]).

Dimba Cave has abundant water and contains several ponds, with average diameters of 3 m and depths of about 1 m, which are located at approximately 400 m from the entrance of the cave (Figure 2).

The cave ponds are fed by the hydrological cycle of the karst system. This karst has morphological and hydrodynamic zonation that is mainly organized vertically and allows differentiating four layers in the karst aquifer: the epi-karst, the infiltration zone, the pinnate zone, and the drowned zone. The epi-karst is the superficial layer of the limestone formation that is relatively thick (with a few meters to a few tens of meters thickness) and collects surface waters into the karst. The infiltration zone temporarily stores the water and gradually transfers it to the deeper pinnate and drowned zones.

### 2.2. Sampling

Water and sediment sampling were conducted at three ponds in Dimba Cave on August 2021. Ponds were numbered 1, 2, and 3 in sequence from the nearest to the furthest to the cave entrance. Three water samples of 250 mL each were collected from ponds and labelled 1A, 1B, 1C for the first pond, 2A, 2B, 2C for the second pond, and 3A, 3B, 3C for the third pond. These samples were directly collected with clean and sterilized polypropylene bottles. The water samples for heavy metal analysis were acidified with 1% *v*/*v* HNO_3_. Replicate water samples for microbiological analysis were also collected with sterilized bottles and were not acidified [19].

In the same ponds, five samples of bottom sediments were collected in each pond. These samples, each with approximately 250 g from the sediment surface layer (0–3 cm), were manually collected at 1 m from the shore and at a depth of 0.5–1 m. These 15 sediment samples were labelled 1S1, 1S2, 1S3, 1S4, and 1S5 for the first pond, 2S1, 2S2, 2S3, 2S4, and 2S5 for the second pond, and 3S1, 3S2, 3S3, 3S4, and 3S5 for the third pond. After collection, all samples were packaged and preserved at 4 °C.

Sediment and water samples were sent to University of Geneva for heavy metals’ analysis. Microbial analyses were carried out in the microbiological laboratory of the University of Kinshasa, Chemistry Department.

### 2.3. Physicochemical Parameters

In situ measurements of pond water parameters, including temperature (T), electrical conductivity (EC), and hydrogen potential (pH), were performed using a multiparameter probe HQ40D from HACH (Auckland, New Zeland).

The grain size of sediment particles was determined on aliquots of about 1 g of fresh sediment from the ponds. Following 5 min of ultrasonic sediment dispersion in deionized water, particle grain size was determined with a Coulter LS-100 Laser diffractometer (Beckman Coulter, Fullerton, CA, USA) [14].

Sediment water content (WC) was determined based on the weight loss after sediment drying in an oven at 100 °C to constant weight. Sediment organic matter (OM) content was determined based on the weight loss of dry sediment aliquots after 1 h of combustion at 550 °C in a muffle furnace (Salvis AG, Lucerne, Switzerland) [20].

### 2.4. Sediment and Water Samples Treatment for Metal Analysis

After freeze-drying, the sediment samples were ground to a fine powder, homogenized, and sieved using a 63 μm sieve to remove occasional stones and debris. The <63 μm sediment fraction was retained for analysis in weighed aliquots, digested as described in Atibu et al. [21]. In short, a weighed aliquot of about 10–15 mg of sample (<63 µm fraction) was subject to complete digestion in pure mineral acids inside Teflon “bombs” heated on a vitro-ceramic hot plate. Sediment digestion was performed in three steps using (a) 1 mL of HNO_3_ (suprapure, 65%), then (b) 0.5 mL of a HClO_4_ mixture (suprapure, 70%) and 0.5 mL of HF (suprapure, 40%), (c) and, finally, 0.5 mL of HNO_3_ (suprapure, 65%). Between each step, the solvents were evaporated to dry residue, and the residue dissolved with the next treatment. The residue from the final step was dissolved in 10 mL of a 1% HNO_3_ solution, and the metal analysis was performed within 24 h.

For water samples, 10 mL of acidified samples was filtered using 0.45 µm membrane filters (Millex^®^-LCR, Millipore, Darmstadt, Germany) to remove particulate matter. A solution of 1% HNO_3_ was used for specific dilutions prior to metal analysis.

### 2.5. Analysis of Heavy Metals in Sediment and Water Samples

The concentrations of heavy metals in the samples were determined by inductively coupled plasma mass spectrometry using the Agilent 7700 × ICP-MS series developed for the analysis of complex matrices. To avoid spectral interferences, a helium mode and specific interference equations were used. Calibration was performed using multi-element standard solutions at different concentrations (0, 0.02, 1, 5, 20, 100, and 200 mg L^−1^) [22].

The limit of detection (LOD) was determined for each element as three times the standard deviation of the blank (Table 1). The concentration of each metal and associated analytical uncertainty were determined as the average and standard deviation of triplicate analysis of the same sample. The relative standard errors of metal concentrations were generally less than 10%, while the procedural chemical blanks were less than 2% of the sample signal. The results were expressed in mg L^−1^ and mg kg^−1^ (dry weight) for water and sediment samples, respectively.

### 2.6. Analysis of Total Mercury in Sediment Samples

An atomic absorption spectrometer Advanced Mercury Analyzer (AMA 254, Altec s.r.l., Czech Republic) was used for the quantitative determination of total mercury in the samples. The method of analysis consisted in the combustion of sediments, followed by the fusion of mercury in a gold trap and, finally, by the measurement of gaseous mercury with the AMA. The detection limit, determined as three times the standard deviation of the blank, was 0.005 mg kg^−1^ (Table 1) [23].

### 2.7. Analysis of Total Mercury in Water Samples

Cold vapor atomic fluorescence spectrometry (CVAFS) (Merx Model III, Brooks Rand, Seattle, WA, USA) was used for the analysis of total mercury (THg) in water samples. Sample preparation was carried out according to the procedure described by Gallorini and Loizeau [24]. This procedure is similar to that of EPA 1631 [25]. It consists of weighing 100 mL of a pre-acidified water sample with 0.5 mL of HNO_3_ and adding 0.5 mL of SnCl_2_ at 20% to reduce Hg(II) to volatile mercury Hg(0). Oxygen was removed by purging the sample with argon for 20 min at a flow rate of 350 mL min^−1^, while the gold trap was purged with argon for 5 min at a flow rate of 60 mL min^−1^. THg was then measured using CVAFS. The detection limit, calculated as three times the standard deviation of the blank, was 0.20 ng L^−1^.

### 2.8. Analytical Quality Control

The reliability of the analytical procedure, the sensitivity of the device and trueness of the results were tested for water and sediment matrices through the repeated analyses of the certified reference materials TMDA-70 and LKSD4 (Environment Canada), respectively (Table 1). Certified reference materials (CRM) were selected to match the matrix of the samples from Dimba Cave. The metal concentrations determined in the CRMs were in excellent agreement with the CRM certified reference values (Table 1).

### 2.9. Assessment of Sediment Pollution

Sediment pollution was evaluated by calculating two parameters, the enrichment factor (EF) and the geoaccumulation index (Igeo) [26]. In order to detect metal pollution from anthropogenic origin and discriminate it from the natural geochemical background, the EF was calculated as follows:

EF = [metal/Sc]_sample_/[metal/Sc]_background_ (1)

where “metal” is the heavy metal concentration in the sediment sample or in the geochemical background and Sc is the scandium concentration in the sediment sample or in the geochemical background. Sc was used to carry out a normalization of analytical results, and the Upper Continental Crust (UCC) metal concentrations were considered as the geochemical background concentrations for the investigated heavy metals [27].

The degree of pollution was assessed by calculating the Igeo as follows:

Igeo = Log_2_[C_m_]/1.5[B_m_] (2)

where C_m_ is the concentration of metal (m) in the sediment, B_m_ is the concentration of the same metal (m) in the geochemical background, and 1.5 is the correlation matrix safety factor to account for the variation of the natural geochemical background [20].

### 2.10. Ecological Risk Parameters

To quantify the heavy metal concentration in sediment samples, the contamination factor (CF) was calculated as follows [28,29]:

CF = (C_n_/B_n_) (3)

where C_n_ is the concentration of heavy metals in the sediment sample and B_n_ is the concentration of heavy metals in the geochemical background.

The assessment of the polymetallic contamination level for each sediment sample was carried out by calculating the contamination degree (CD) as follows [30]:

CD = ΣCF_i_ (4)

where i stands for a specific heavy metal and CF_i_ is the contamination factor for the heavy metal i. The heavy metals Hg, Cd, As, Co, Cu, Pb, Cr, and Zn were considered in the CD calculation.

The ecological risk index (E_ri_) was calculated to assess the harmful impact of heavy metals on both the environment and human health. This parameter reflects the ecological sensitivity and the toxicity of pollutants [31]. E_ri_ was determined as follows:

E_ri_ = Tr_i_ × CF_i_ (5)

where Tr_i_ represents the response factor to a given toxic heavy metal or biological toxic factor of the heavy metal. Tr_i_ values used for each element (in parenthesis) were 40 (Hg), 30 (Cd), 10 (As), 5 (Co), 5 (Cu), 5 (Pb), 2 (Cr), and 1 (Zn) [32]. CF_i_ is the contamination factor for the heavy metal i.

The potential ecological risk index (RI) for polymetallic contamination in each sample was calculated by summing the singular ecological risk indices. This RI factor takes into account the synergy between the toxic level, the concentration of heavy metals, and the ecological sensitivity of biological communities to heavy metals [33]. The RI was calculated as follows:

RI = ΣE_ri_ (6)

where E_ri_ is the ecological risk index for the heavy metal i.

The ecotoxicological risk posed by metal pollutants in sediments was also assessed through comparison of metal concentrations determined in Dimba Cave sediments against the limits recommended in Sediment Quality Guidelines (SQG) for the protection of aquatic life and the Probable Effect Levels (PEL) on aquatic biota, as adopted by Canada [34].

### 2.11. Microbial Analysis in Water Samples

The quantification of microbial populations such as those of *Escherichia coli* (*E. coli*), enterococci (ENT), total coliforms (TC), and *Pseudomonas* spp. (*P*. spp.) was performed using the membrane filtration method. In brief, after filtration of water samples through 47 mm diameter and 0.45 μm pore-size membrane sterile filters (Millipore, Bedford, MA, USA), the filters were placed on selective culture media (Biolife, Italy) supplemented with the antifungal compound Nystatin (100 μg mL^−1^ final concentration).

The following incubation conditions were used: *E. coli*: Tryptone Soy Agar (TSA) medium, incubated at 37 °C for 4 h and transferred to Tryptone Bile X-Gluc Agar (TBX) medium at 44 °C for 24 h; ENT: Slanetz Bartley Agar (SBA) medium, incubated at 44 °C for 48 h and transferred into Bile Aesculin Agar (BAA) medium at 44 °C for 4 h; TC: Endo Agar, incubated at 35 °C for 24 h; *P*. spp.: *Pseudomonas* selective Agar (PSA), incubated at 37 °C for 24 h.

The number of colony-forming units per 100 mL of water (CFU 100 mL^−1^) was used to express the results. The reproducibility of the whole experimental procedure was tested by means of triplicate analyses of selected samples. The results of triplicate analysis displayed a mean coefficient of variation of 8% for *E. coli* and 9% for both ENT and TC [35,36].

### 2.12. Data Treatment

Triplicate determinations of heavy metal concentrations were carried out on each sample and averaged. Statistical data treatment (Spearman correlation) was performed using SigmaStat 11.0 software (Systat Software, Inc., San Jose, CA, USA) and XLSTAT software version 2021.1 from Addinsoft [37] (Statistical and data analysis solution, New York, United States; https://www.xlstat.com, accessed on 3 June 2024).

## 3. Results and Discussion

### 3.1. Physicochemical Characteristics of Water and Sediment Samples

Table 2 shows the results of determination of water (Table 2a) and sediment (Table 2b) physicochemical parameters, including the temperature (T), hydrogen potential (pH), electrical conductivity (EC), water content (WC), organic matter (OM), and mean size of sediment particles.

For the three ponds investigated in Dimba Cave, the water temperature ranged from 23 to 24 °C. These values were within the 12–25 °C range set by the WHO for drinking water of acceptable quality. Dimba Cave waters were acidic, with pH values in the range of 3.25–4.44, and were well below the WHO range of pH recommended values (6.5–9.5) for drinking water of acceptable quality [38]. Acidic groundwater in limestone lithology is unusual and may indicate intrusion of acidic and contaminated water from waste releases into the environment and/or contamination of the ponds by miners and bat guano collectors. The EC values in Dimba Cave water samples varied from 312 to 444 µS cm^−1^ and were within the range recommended by the WHO (200–800 µS cm^−1^) for drinking water [38].

The sediment water content (WC) ranged from 48 to 60% for all samples, while organic matter (OM) ranged from 6.34 to 7.91% of sediment dry weight (Table 2b). The mean size of sediment particles ranged from 4.72 to 10.95 μm for all samples. According to the descriptive terminology adopted in the GRADISTAT program, the sediment samples consisted mostly of medium and fine silts [39].

### 3.2. Heavy Metal Concentrations in the Water Samples

The concentration values of heavy metals in the water samples are reported in Table 3. Pond 3 showed the highest metal concentrations, but in the three ponds, almost all samples showed very high concentrations of Cr, Mn, Ni, Cu, As, Cd, Pb, and Hg that practically exceeded the limit values set for drinking water [40]. The concentration levels of other metals, namely Sc, Ti, V, Fe, Co, Zn, and Ba, were often elevated as well but not exceeding the permissible limits for drinking water. Limits recommended by the WHO for heavy metals in drinking water were included for the elements available in Table 3, as were the limits adopted by the European Union for drinking water quality [41]. From the comparison of concentrations, several heavy metals in these pond waters do not allow its use as drinking water for human consumption.

### 3.3. Concentrations of Heavy Metals in the Sediment Samples

Table 4 shows the results of heavy metal analysis in sediment samples. The highest concentration values in the samples were 15.2 mg kg^−1^ (Sc), 270 mg kg^−1^ (Ti), 72.3 mg kg^−1^ (V), 62.0 mg kg^−1^ (Cr), 10,430 mg kg^−1^ (Mn), 46,698 mg kg^−1^ (Fe), 15.3 mg kg^−1^ (Co), 57.4 mg kg^−1^ (Ni), 339 mg kg^−1^ (Cu), 1065 mg kg^−1^ (Zn), 6.1 mg kg^−1^ (As), 1.2 mg kg^−1^ (Cd), 1023 mg kg^−1^ (Ba), 24.6 mg kg^−1^ (Pb), and 1.2 mg kg^−1^ (Hg).

Most sediments’ concentrations of Cr, Cu, Zn, As, Cd, and Hg exceeded the sediment quality guideline values (SQG) for the protection of aquatic life [34]. Concentrations even higher than SQG values and exceeding the Probable Effect Levels (PEL) for aquatic biota were determined in almost all samples for Cr, Cu, Zn, Cd, and Hg, with Hg and Zn displaying concentrations 2.5 to 3.5 times higher than their respective PEL values. Therefore, the sediments from cave ponds were contaminated with heavy metals in concentrations largely exceeding the sediment quality guidelines. The current contamination levels of these sediments may put in danger the aquatic biota of the cave’s ecosystem [42].

### 3.4. Estimation of the Pollution Level

In order to discriminate heavy metals of anthropogenic origin from metals of geogenic origin, the EF and Igeo indices were calculated and used to build a criterion (a scale) to classify the level of sediment pollution by heavy metals [43]. The results are presented in Table 5 and Table 6, respectively.

The highest EF values of Hg (2 samples) and Cu (1 sample) were between 25 and 50, indicating “very severe enrichment” of this metal in sediment. Other samples showed “severe enrichment” for Hg and “severe enrichment” to “moderately severe enrichment” for Cu. The EF values of Cr, Mn, Co, Ni, Zn, As, and Cd varied across sampling sites, ranging from “minor enrichment” to “severe enrichment”, while “no enrichment” was observed for Pb in the samples from Ponds 2 and 3, and a “minor enrichment” for the samples from Pond 1. On the one hand, according to the calculated Igeo values, all the samples analyzed were “heavily polluted” by Hg, while only some samples collected in Pond 2 were “heavily polluted” by Mn, Cu, Zn, and Cd (Table 5). On the other hand, all the samples were “practically unpolluted” by Co and Pb and ranked as “unpolluted to moderately polluted” by Cr and Ni. Regarding Mn, Cu, Zn, As, and Cd, the samples were classified from “unpolluted to moderately polluted” and from “moderately to heavily polluted”.

Therefore, although there was a wide range of contamination levels for individual metals, globally, the pond sediments were highly contaminated by several toxic metals.

### 3.5. Statistical Correlations

The Spearman rank correlation was applied to parameters measured in water and sediment samples. The results are shown in Table 7 and Table 8, respectively.

A correlation analysis of concentrations of several heavy metals with temperature (T), pH, and electrical conductivity (EC) was carried out, and the results are reported in Table 7. The results indicated strong and positive correlations (*p* < 0.05) between T and V, Mn, Fe, Co, As, and Hg, while negative correlations were observed between pH and V, Cr, Fe, Ni, Zn, As, Pb, and Hg. EC also had negative correlations with V, Cr, Mn, Fe, Co, Ni, Zn, As, Pb, and Hg. In general, strong and positive correlations were observed between all heavy metals, except between Cd and V, Cr, Mn, Fe, Co, and Pb ; between Hg and Cd; between Pb and V, Fe, and Co ; between Zn and V and Fe; and between Cu and V, Mn, Fe, and Co, for which no correlations were observed. The strong and positive correlations suggested that the heavy metals came from a common source and were transported into the ponds’ water by the same route. Additionally, the positive correlation would indicate that T had a positive impact on this transport. The negative correlation between heavy metals, pH, and EC indicated that the migration of heavy metals was negatively influenced by these parameters [44,45].

Regarding the sediment matrix, a correlation analysis between several heavy metals, WC, OM, and grain size was performed, and Table 8 shows the obtained results. The positive correlations between heavy metals and WC, OM, and grain size indicated that the accumulation of heavy metals in the sediments would be influenced by OM, WC, and grain size. Positive correlations were also observed between metals such as Cr and Pb (r = 0.587); Mn and Ni (r = 0.811), Cu (r = 0.800), Zn (r = 0.979), As (r = 0.782), and Hg (r = 0.926); Co and Ni (r = 0.500) and Cu (r = 0.581); Ni and Cu (r = 0.791), Zn (r = 0.808), As (r = 0.850), and Hg (r = 0.847); Cu and Zn (r = 0.814), As (r = 0.665), Cd (r = 0.612), and Hg (r = 0.786); Zn and As (r = 0.814) and Hg (r = 0.926); As and Hg (r = 0.815). These correlations suggest that these metals may have originated from the same sources and were transported together [45].

### 3.6. Ecological Risk Parameters

Table 9 shows the results of the ecological risk parameters, including the contamination factor (CF), the potential ecological risk factor (E_ri_), the contamination degree (CD), and the ecological risk index (RI). The CF showed that, in general, the sediments were “considerably contaminated” (3 < CF < 6) or “very strongly contaminated” (6 < CF) by Cu, Zn, As, Cd, and Hg. Some samples displayed “moderate contamination” (1 < CF < 3) or “low contamination” (CF < 1) with Cr, Co, As, and Pb. Concerning the CD values, all the sediment samples showed “very high contamination” (32 ≤ CD).

It was noticed that the sediment samples had low ecological risks (Eri < 40) or moderate ecological risks (40 < Eri < 80) for Cr, Co, Cu, Zn, As, and Pb, whereas high ecological risks (160 < Eri < 320) or very high ecological risks (Eri > 320) were detected for Cd and Hg. Regarding the RI parameter, all the samples showed very high ecological risks or a serious ecological pollution level (IR > 600).

In general, these parameters indicated that anthropic activities, including activities of local populations, resulted in serious contamination of the cave environment. This contamination may have occurred at the surface and reached the cave through water infiltration in the karst, but might have occurred also in the cave from activities such as guano and lime exploitation.

### 3.7. Microbiological Parameters of Water Samples

Table 10 shows the population densities of *Escherichia coli* (*E. coli*), *Enterococcus* spp. (*ENT*), total coliforms (TC), and *Pseudomonas* spp. (*P*. spp.) determined in the water samples from Dimba Cave ponds. To assess the sanitary quality of water, the European Union (EU) and the United States Environmental Protection Agency (USEPA) recommend the use of *E. coli* (a fecal coliform) and *ENT* (the members of *Enterococcus* genus) as indicators [46,47].

The microbial population densities in Dimba Cave pond waters varied with the sampling sites between 123 and 243 CFU 100 mL^−1^ for *E. coli*, 8 and 16 CFU 100 mL^−1^ for ENT, 20 and 28 CFU 100 mL^−1^ for TC, and 10 and 16 CFU 100 mL^−1^ for *P*. spp. These values clearly showed microbiological contamination of the waters of Dimba Cave, and, according to the European Directive (EU) 2020/2184 [41] for drinking water quality, the cave water is not suitable for human consumption [47].

Spearman rank correlation coefficients applied to microbiological parameters measured in water samples are shown in Table 11.

A positive and significant correlation (*p* < 0.05) was observed between *E. coli*/TC (r = 1). This is understandable because *E. coli* is a subset of the fecal coliform group. Significant correlations were observed between *P.* spp. (a non-fecal indicator bacteria) and *E. coli* (r = 0.5) and TC (r = 0.5), suggesting an origin other than fecal matter. Negative and significant correlations between *E. coli*/*ENT* (r = −0.5), *ENT*/TC (r = −0.5), and *ENT/P.* spp. (r = −1) suggested that they originated from different sources that could be either a natural source or human or animal fecal matter and that they were transported by different pathways [48].

To better define the natural or non-natural origin of *E. coli* and *ENT*, the *E. coli* and *ENT* genomic profiles of general origin should be examined by PCR tests using primers and specific operating conditions [48,49,50].

## 4. Conclusions

This work allowed obtaining the first information on heavy metal concentrations and related ecotoxicological risks, and on the microbial contamination of pond waters and sediments from Dimba Cave. The results underpin several conclusions, as follows.

Most water samples contained very high concentrations of V, Cr, Mn, Fe, Ni, Cu, Zn, As, Cd, Pb, and Hg, all exceeding the maximum permissible values set in the WHO recommendations for waters used for human consumption. Therefore, the use of Dimba Cave water by local populations as a drinking water is a threat to public health and must be discouraged. Because of the water–food–human health nexus, the use of this water for cooking and irrigation may also add metal contaminants to the diet and, therefore, should be discouraged.

In general, all sediment samples showed higher concentrations of Cr, Cu, Zn, Cd, and Hg when compared with the sediment quality guidelines for the protection of aquatic life.

In the majority of sediment samples, the enrichment factor and the geoaccumulation index parameters indicated serious pollution by heavy metals that possibly originated from anthropogenic activities. Nevertheless, further research shall be carried out to identify the pollution sources and to clarify whether the limestone could also be a source of these metals.

The calculated ecological risk parameters, including contamination factor, ecological risk factor, contamination degree, and ecotoxicological risk index, indicated that sediments may pose a very high ecotoxicological risk to the aquatic cave biota that, in general, is very fragile.

The microbiological analyses of pond waters revealed the presence of *Escherichia coli*, *Enterococcus*, total coliforms, and *Pseudomonas* spp. The presence of fecal coliform bacteria indicated strong contamination by human and/or animal manure. The Spearman correlation (*p* < 0.05) of these microbial parameters suggested different origins and pathways of microbial contamination, which could be anthropogenic sources at the surface and exploitation of bat guano in the cave.

Globally, this investigation showed that the Dimba Cave environment that was sealed from human intrusion until the 1920s is now highly polluted by toxic heavy metals and pathogenic bacteria and, thus, the groundwater from the cave is not suitable for human consumption.

The results of this work provide a sound basis for action by decision makers to enact Dimba Cave management to improve water quality and to protect the cave environment and public health, contributing also to the preservation of this candidate to UNESCO World Heritage. The improved management of Dimba cave and groundwater protection measures could also contribute to attaining the UN Sustainable Development Goals and meeting the objectives of the 2030 Agenda for Sustainable Development [51].

## Figures and Tables

**Table 1:** Limits of detection (LOD) for the analyzed elements, and results of the determination of metals in lake water and sediment certified reference materials (CRM). Triplicate laboratory determinations of individual elements in CRMs displayed relative standard deviations generally lower than 5%. Certified values of CRMs are included for comparison. “–”: CRM value not available or not determined.

Sample	Sc	Ti	V	Cr	Mn	Fe	Co	Ni	Cu	Zn	As	Cd	Ba	Pb	THg
LOD (mg kg^−1^)	–	0.098	0.024	0.000	0.150	0.681	0.025	0.000	1.516	2.033	0.049	0.063		1.091	0.005
CRM TMDA-70 (µg L^−1^):															
Determined value	–	0.47	315	386	306	366	284	331	389	481	41	143	–	442	–
Certified value	–	0.39	312	388	302	368	285	328	399	480	40	145	309	443	–
CRM LKSD4 (mg kg^−1^):															
Determined value	–	–	31.2	19.6	427	–	10.2	31.6	29.5	179.3	11.4	1.6	–	92.7	–
Certified value	–	–	32	21	430	–	11	32	30	189	12	1.9	–	93	–
CRM MESS-4 (mg kg^−1^):															
Determined value	–	–	–	–	–	–	–	–	–	–	–	–	–	–	0.081 ± 0.009
Certified value	–	–	–	–	–	–	–	–	–	–	–	–	–	–	0.09 ± 0.04

**Table 2:** Physicochemical parameters determined in Dimba Cave samples.

**a: Physicochemical Parameters of the Water Samples**				
**Site**	**Sample**	**T (°C)**	**pH**	**EC (µs cm^−1^)**
	1A	24	3.96	399
Pond 1	1B	23	4.22	405
	1C	23.5	4.44	439
	2A	23	3.92	395
Pond 2	2B	22.9	4.23	407
	2C	23.3	4.4	444
	3A	23.5	3.25	312
Pond 3	3B	24	3.45	340
	3C	24.3	4.01	401
WHO recommendation *		12–25	6.5–9.5	200–800
**b: Physicochemical Parameters of the Sediment Samples**				
**Site**	**Sample**	**WC** **(%)**	**OM** **(%)**	**Mean Grain Size (μm)**
	1S1	50.46	6.48	6.29
	1S2	48.18	7.10	5.31
Pond 1	1S3	49.25	6.64	8.09
	1S4	50.47	6.34	8.54
	1S5	51.59	6.60	4.72
	2S1	56.02	7.72	8.85
	2S2	55.18	7.54	10.49
Pond 2	2S3	56.89	7.54	9.27
	2S4	54.16	7.48	10.95
	2S5	55.74	7.91	10.38
	3S1	59.95	7.32	6.24
	3S2	58.40	7.85	8.71
Pond 3	3S3	59.18	7.78	7.82
	3S4	52.36	6.77	5.75
	3S5	52.69	6.88	6.88

* Limit set by World Health Organization Guidelines for Drinking Water Quality [38].

**Table 3:** Heavy metal content (mg L^−1^) in filtered water samples from Dimba Cave ponds. The heavy metal concentration values that exceeded the recommended limit values set by the WHO for drinking water are shown in bold [40].

Site	Sample	Sc	Ti	V	Cr	Mn	Fe	Co	Ni	Cu	Zn	As	Cd	Ba	Pb	Hg
	1A	0.2	27.4	7.9	**4**	**38.4**	8872	0.2	bdl	bdl	15	**0.1**	bdl	33.5	bdl	**0.05**
Pond 1	1B	0.4	31.2	9	**47**	**54.3**	9273	0.3	bdl	bdl	18.8	**0.2**	bdl	36.5	bdl	**0.06**
	1C	0.2	26.6	7.7	**4**	**40.1**	8488	0.2	bdl	bdl	13.9	**0.1**	bdl	33	bdl	**0.05**
	2A	0.1	36.6	4.7	**4.1**	**23.8**	3932	bdl	**0.1**	**8.9**	67	**0.1**	bdl	20.8	**9.0**	**0.06**
Pond 2	2B	0.1	37.8	5.3	**4.7**	**23.9**	4238	0.1	**0.9**	**12.8**	81.1	**0.1**	**2.2**	24.1	**6.9**	**0.07**
	2C	bdl	31.7	3.9	**3.5**	**14.1**	3133	bdl	bdl	**5.5**	57.9	bdl	bdl	13.4	**3.4**	**0.04**
	3A	4.7	79	61.2	**31.6**	**146.3**	33,293	1.8	**5.0**	**11.5**	111.2	**1.4**	bdl	67.4	**18.3**	**0.46**
Pond 3	3B	3.6	89.6	38.9	**29.4**	**208.1**	27,145	2.6	**7.1**	**26.9**	249.7	**1.6**	**0.2**	99.7	**47.9**	**0.51**
	3C	4	81.1	46	**29.3**	**180.1**	28,715	2.3	**6.1**	**21.3**	198.1	**1.5**	**0.1**	88.7	**35.1**	**0.51**
WHO recommended limit					0.05	0.4			0.07	2		0.01	0.003		0.01	0.006
EU adopted limit					0.025	0.05	0.2	2.0	0.020						0.005	0.001

bdl, below detection limit.

**Table 4:** Heavy metal concentrations in sediment samples (mg kg^−1^ dry weight) from Dimba Cave ponds. The heavy metal concentration values exceeding the Sediment Quality Guidelines for the Protection of Aquatic Life are shown in bold [34]. SQG—sediment quality guidelines, PEL—probable effect levels.

Site	Sample	Sc	Ti	V	Cr	Mn	Fe	Co	Ni	Cu	Zn	As	Cd	Ba	Pb	Hg
	1S1	5.7	182	64.9	**58.6**	2868	30,010	15.2	49.1	**258**	**489**	4.2	**0.7**	466	22	**0.7**
	1S2	4.5	156	59.4	**53.9**	2522	24,584	13.2	42.8	**228**	**456**	3.8	**0.8**	429	20.4	**0.7**
Pond 1	1S3	10.2	237	63.7	**56.1**	2852	35,229	14.7	47.3	**248**	**472**	5	**0.7**	744	20.7	**0.7**
	1S4	4.3	168	68.2	**61.3**	2797	26,245	14.2	48.1	**251**	**486**	4.2	**0.7**	473	21.1	**0.7**
	1S5	6.9	194	65.7	**58.8**	2867	30,626	14.8	48.1	**250**	**486**	4.4	**0.7**	453	21.3	**0.7**
	2S1	14.4	265	72.3	**60.8**	7244	44,156	14.6	54.5	**277**	**898**	5.8	**0.9**	643	24.6	**1.2**
	2S2	15.2	256	71.6	**60.9**	7932	45,541	14.8	55.7	**312**	**926**	**6.1**	**0.8**	686	21.2	**1.2**
Pond 2	2S3	13.6	270	71.4	**58.6**	9675	41,818	14.4	54.4	**298**	**997**	5.7	**1.0**	947	19.5	**1.2**
	2S4	14.3	228	69.2	**57.7**	8155	44,242	15.2	55.7	**294**	**859**	5.6	**0.8**	759	21	**1.2**
	2S5	14.1	258	69.2	**56.5**	10430	44,592	15.3	57.4	**339**	**1065**	5.8	**1.2**	1023	19.7	**1.2**
	3S1	14.8	220	69.9	**62**	5755	46,698	14.7	57.1	**265**	**719**	5.8	0.6	580	22.1	**1**
	3S2	14.2	202	67.5	**60.7**	4095	45,080	14.2	53.9	**244**	**676**	5.3	0.5	473	20.4	**1**
Pond 3	3S3	14.6	232	68.1	**59.5**	5912	43,867	14.8	54.7	**252**	**763**	5.7	**0.7**	566	19.9	**1**
	3S4	13.8	253	66.9	**57.6**	6044	42,319	14	54.4	**257**	**821**	5.6	0.6	553	18	**1**
	3S5	13.4	255	67.4	**56.9**	5938	42,492	14.4	53.7	**241**	**781**	5.7	0.6	552	18.1	**0.9**
	SQG				37.3					35.7	123	5.9	0.6		35	0.17
	PEL				90					197	315	17	3.5		91.3	0.486

**Table 5:** Enrichment factor (EF) values of heavy metals in sediment samples from Dimba Cave ponds.

Sample	Cr	Mn	Co	Ni	Cu	Zn	As	Cd	Pb	Hg
**1S1**	3.2	9.2	2.9	4.7	19.9	13.3	5.4	13.8	2.1	24.1
**1S2**	3.8	1.3	3.2	5.2	22.2	15.7	6.2	20	2.5	30.6
**1S3**	1.7	5.1	1.4	2.6	10.7	7.2	3.6	7.7	1.1	13.5
**1S4**	4.5	11.9	3.6	6.2	25.6	17.5	7.2	18.3	2.7	32
**1S5**	2.7	7.6	2.4	3.8	15.9	10.9	4.7	11.4	1.7	19.9
**2S1**	1.3	9.2	1.1	2.1	8.5	9.7	3	7	0.9	16.4
**2S2**	1.3	9.6	1.1	2	9	9.4	2.9	5.9	0.8	15.5
**2S3**	1.4	13	1.2	2.2	9.6	11.4	3.1	8.3	0.8	17.3
**2S4**	1.3	10.5	1.2	2.1	9	9.3	2.9	6.3	0.8	16.5
**2S5**	1.3	13.6	1.2	2.2	10.6	11.7	3	9.6	0.8	16.7
**3S1**	1.3	7.1	1.1	2.1	7.9	7.5	2.9	4.6	0.8	13.3
**3S2**	1.3	5.3	1.1	2.1	7.6	7.4	2.7	4	0.8	13.8
**3S3**	1.3	7.4	1.1	2.1	7.6	8.1	2.9	5.4	0.7	13.5
**3S4**	1.3	8	1.1	2.2	8.2	9.2	3	4.9	0.7	14.2
**3S5**	1.3	8.1	1.2	2.2	7.9	9	3.1	5	0.7	13.2
**Geochemical background values** **(mg kg^−1^) [27])**	35	600	10	20	25	71	1.5	0.098	20	0.056
EF interpretation values:										
EF < 1: no enrichment										
EF 1–3: minor enrichment										
EF 3–5: moderate enrichment										
EF 5–10: moderate severe enrichment										
EF 10–25: severe enrichment										
EF 25–50: extremely severe enrichment										

**Table 6:** Igeo values of heavy metals in sediment samples from Dimba Cave ponds.

Sample	Cr	Mn	Co	Ni	Cu	Zn	As	Cd	Pb	Hg
**1S1**	0.2	1.7	0	0.7	2.8	2.2	0.9	2.3	−0.4	3.1
**1S2**	0	1.5	−0.2	0.5	2.6	2.1	0.8	2.4	−0.6	3.1
**1S3**	0.1	1.7	0	0.7	2.7	2.1	1.2	2.3	−0.5	3.1
**1S4**	0.2	1.6	−0.1	0.7	2.7	2.2	0.9	2.3	−0.5	3.1
**1S5**	0.2	1.7	0	0.7	2.7	2.2	1	2.3	−0.5	3.1
**2S1**	0.2	3	0	0.9	2.9	3.1	1.4	2.6	−0.3	3.8
**2S2**	0.2	3.1	0	0.9	3.1	3.1	1.4	2.4	−0.5	3.8
**2S3**	0.2	3.4	−0.1	0.9	3	3.2	1.3	2.8	−0.6	3.8
**2S4**	0.1	3.2	0	0.9	3	3	1.3	2.4	−0.5	3.8
**2S5**	0.1	3.5	0	0.9	3.2	3.3	1.4	3	−0.6	3.8
**3S1**	0.2	2.7	0	0.9	2.8	2.8	1.4	2	−0.4	3.6
**3S2**	0.2	2.2	−0.1	0.8	2.7	2.7	1.2	1.8	−0.6	3.6
**3S3**	0.2	2.7	0	0.9	2.7	2.8	1.3	2.3	−0.6	3.6
**3S4**	0.1	2.7	−0.1	0.9	2.8	2.9	1.3	2	−0.7	3.6
**3S5**	0.1	2.7	−0.1	0.8	2.7	2.9	1.3	2	−0.7	3.4
Igeo classification					Colour					
Igeo ≤ 0, Class 0: practically unpolluted										
0 < Igeo < 1, Class 1: unpolluted to moderately polluted										
1 < Igeo < 2, Class 2: moderately polluted										
2 < Igeo < 3, Class 3: moderately to heavily polluted										
3 < Igeo < 4, Class 4: heavily polluted										

**Table 7:** Spearman rank order correlation for physicochemical parameters analyzed in water samples from Dimba Cave. Significant coefficients, with *p* < 0.05, are indicated in bold.

Variable	V	Cr	Mn	Fe	Co	Ni	Cu	Zn	As	Cd	Pb	Hg
T	**0.599**	0.226	**0.633**	**0.599**	**0.668**	0.357	0.24	0.262	**0.524**	0.065	0.343	**0.585**
pH	**−0.583**	**−0.689**	−0.467	**−0.583**	−0.471	**−0.618**	−0.458	**−0.6**	**−0.618**	−0.05	**−0.627**	**−0.689**
EC	**−0.633**	**−0.714**	**−0.533**	**−0.633**	**−0.521**	**−0.618**	−0.424	**−0.55**	**−0.661**	−0.05	**−0.593**	**−0.745**
V		**0.824**	**0.917**	**1**	**0.924**	**0.574**	0.356	0.483	**0.888**	0.168	0.441	**0.894**
Cr			**0.79**	**0.824**	**0.771**	**0.838**	**0.692**	**0.79**	**0.891**	0.464	**0.727**	**0.902**
Mn				**0.917**	**0.992**	**0.644**	0.458	**0.533**	**0.957**	0.347	**0.509**	**0.894**
Fe					**0.924**	**0.574**	0.356	0.483	**0.888**	0.168	0.441	**0.894**
Co						**0.628**	0.453	**0.538**	**0.944**	0.349	0.496	**0.874**
Ni							**0.956**	**0.957**	**0.773**	**0.703**	**0.956**	**0.847**
Cu								**0.966**	**0.602**	**0.796**	**0.948**	**0.663**
Zn									**0.696**	**0.673**	**0.966**	**0.727**
As										0.419	**0.664**	**0.934**
Cd											**0.574**	0.410
Pb												**0.739**

**Table 8:** Spearman rank order correlation for physicochemical parameters analyzed in sediment samples from Dimba Cave ponds. Significant coefficients, with *p* < 0.05, are shown in bold.

Variable	Cr	Mn	Co	Ni	Cu	Zn	As	Cd	Pb	Hg
**WC**	**0.542**	**0.568**	0.162	**0.766**	0.439	**0.586**	**0.737**	−0.029	−0.007	**0.692**
**OM**	0.08	**0.636**	0.185	**0.676**	0.413	**0.634**	**0.66**	0.336	−0.206	**0.779**
**Grain size**	0.152	**0.671**	0.358	**0.544**	**0.646**	**0.686**	**0.505**	**0.535**	−0.011	**0.748**
**Cr**		0.005	0.034	0.332	0.268	0.113	0.322	−0.196	**0.587**	0.197
**Mn**			0.394	**0.811**	**0.8**	**0.979**	**0.782**	0.469	−0.270	**0.926**
**Co**				**0.5**	**0.581**	0.317	0.322	0.36	0.327	0.279
**Ni**					**0.791**	**0.808**	**0.850**	0.248	0.025	**0.847**
**Cu**						**0.814**	**0.665**	**0.612**	0.172	**0.786**
**Zn**							**0.814**	0.476	−0.241	**0.926**
**As**								0.251	0	**0.815**
**Cd**									0.081	0.484
**Pb**										−0.106

**Table 9:** Values of ecological risk parameters in sediment samples from Dimba Cave for contamination factor (CF), contamination degree (CD), ecological risk index (E_ri_), and potential ecological index (RI).

				CF					CD					Eri					RI
Site	Cr	Co	Cu	Zn	As	Cd	Pb	Hg		Site	Cr	Co	Cu	Zn	As	Cd	Pb	Hg	
**1S1**	1.7	1.5	10.3	6.9	2.8	7.1	1.1	12.5	43.9	**1S1**	3.3	7.6	51.6	6.9	28	214.3	5.5	500	817.2
**1S2**	1.5	1.3	9.1	6.4	2.53	8.2	1	12.5	42.6	**1S2**	3.1	6.6	45.5	6.4	25.3	244.9	5.1	500	836.9
**1S3**	1.6	1.5	9.9	6.7	3.33	7.1	1	12.5	43.6	**1S3**	3.2	7.4	49.5	6.7	33.3	214.3	5.2	500	819.5
**1S4**	1.8	1.4	10	6.9	2.8	7.1	1.1	12.5	43.5	**1S4**	3.5	7.1	50.1	6.9	28	214.3	5.3	500	815.1
**1S5**	1.7	1.5	10	6.8	2.93	7.1	1.1	12.5	43.7	**1S5**	3.4	7.4	50	6.8	29.3	214.3	5.3	500	816.6
**2S1**	1.7	1.5	11.1	12.7	3.87	9.2	1.2	21.4	62.7	**2S1**	3.5	7.3	55.5	12.7	38.7	275.5	6.2	857.1	1256.4
**2S2**	1.7	1.5	12.5	13	4.07	8.2	1.1	21.4	63.5	**2S2**	3.5	7.4	62.4	13	40.7	244.9	5.3	857.1	1234.3
**2S3**	1.7	1.4	11.9	14	3.8	10.2	1	21.4	65.5	**2S3**	3.3	7.2	59.6	14	38	306.1	4.9	857.1	1290.3
**2S4**	1.6	1.5	11.7	12.1	3.73	8.2	1.1	21.4	61.4	**2S4**	3.3	7.6	58.7	12.1	37.3	244.9	5.3	857.1	1226.4
**2S5**	1.6	1.5	13.5	15	3.87	12.2	1	21.4	70.2	**2S5**	3.2	7.7	67.7	15	38.7	367.3	4.9	857.1	1361.7
**3S1**	1.8	1.5	10.6	10.1	3.87	6.1	1.1	17.9	52.9	**3S1**	3.5	7.4	52.9	10.1	38.7	183.7	5.5	714.3	1016.1
**3S2**	1.7	1.4	9.8	9.5	3.53	5.1	1	17.9	49.9	**3S2**	3.5	7.1	48.8	9.5	35.3	153.1	5.1	714.3	976.6
**3S3**	1.7	1.5	10.1	10.7	3.8	7.1	1	17.9	53.8	**3S3**	3.4	7.4	50.4	10.7	38	214.3	5	714.3	1043.5
**3S4**	1.6	1.4	10.3	11.6	3.73	6.1	0.9	17.9	53.5	**3S4**	3.3	7	51.4	11.6	37.3	183.7	4.5	714.3	1013
**3S5**	1.6	1.4	9.6	11	3.8	6.1	0.9	16.1	50.6	**3S5**	3.3	7.2	48.1	11	38	183.7	4.5	642.9	938.6
**Classification CF**								**Classification RI**											
**CF < 1**			Low contamination					**RI < 150**			Low ecological risk or low ecological pollution level								
**1 < CF < 3**			Moderate contamination					**150 ≤ RI < 300**			Moderate ecological pollution level or moderate ecological risk								
**3 < CF < 6**			Considerable contamination					**300 ≤ RI < 600**			Considerable ecological risk or severe ecological pollution level								
**6 < CF**			Very high contamination					**RI > 600**			Very high ecological risk or serious ecological pollution level								
**Classification Eri**								**Classification CD**											
**Eri < 40**			Low ecological risk					**8 ≤ CD < 16**			Moderate contamination								
**40 < Eri < 80**			Moderate ecological risk					**16 ≤ CD < 32**			High contamination								
**80 < Eri < 160**			Considerable ecological risk					**32 ≤ CD**			Very high contamination								
**160 < Eri < 320**			High ecological risk																
**Eri > 320**			Very high ecological risk																

**Table 10:** The population densities (CFU 100 mL^−1^) of *E. coli*, *ENT*, TC, and *P.* spp. in water samples from Dimba Cave. Limit values recommended by the WHO and adopted by the EU for drinking water were included for comparison.

Site	*E. coli*	ENT	TC	*P.* spp.
Pond 1	243	8	28	16
Pond 2	123	11	20	12
Pond 3	163	16	24	10
WHO recommended limit	0	0	0	
EU adopted limit	0	0	0	

**Table 11:** Spearman rank order correlation for microbiological parameters analyzed in water samples. Positive and significant coefficients, with *p* < 0.05, are indicated in bold.

Variables	ENT	TC	*P.* spp.
*E. coli*	−0.5	**1**	**0.5**
ENT		−0.5	−1
TC			**0.5**

## Data Availability

The original contributions presented in the study are included in the article.
